# Lymphocyte-sparing effect of stereotactic body radiation therapy compared to conventional fractionated radiation therapy in patients with locally advanced pancreatic cancer

**DOI:** 10.1186/s12885-019-6220-1

**Published:** 2019-10-22

**Authors:** Guangyin Wu, Michael J. Baine, Nan Zhao, Sicong Li, Xiaobo Li, Chi Lin

**Affiliations:** 1 Department of Radiation Oncology, Henan Provincial People’s Hospital; People’s Hospital of Zhengzhou University, Henan, China; 2 Department of Radiation Oncology, University of Nebraska Medical Center, 986861 Nebraska Medical Center, Omaha, NE 68198-68618 USA; 3 Department of Radiation Oncology, Fujian Medical University Union Hospital, Fuzhou, Fujian China; 4 College of Medical Technology and Engineering, Fujian Medical University, Fuzhou, Fujian China

**Keywords:** Lymphocyte-sparing, SBRT, Pancreatic cancer

## Abstract

**Background:**

Conventionally fractionated (CF) radiation therapy (RT) has been associated with lymphopenia, leading to compromised overall survival (OS) in cancer patients. It currently remains unknown if stereotactic body (SB) RT induces lymphopenia to the same degree. The aim of this study is to determine if SBRT with either chemotherapy (CMT) (Fluorouracil (5FU) or capecitabine) or Nelfinavir (NFV) to pancreatic adenocarcinoma induces lymphopenia to the same degree as CFRT with 5FU or capecitabine and how any associated difference affects patient survival outcomes.

**Methods:**

Medical records of pancreatic adenocarcinoma patients treated with induction CMT followed by RT with concurrent CMT or NFV were reviewed. Patients with total lymphocyte counts (TLCs) available both prior to and following initiation of RT were included. Three groups were identified: CFRT/CMT, SBRT/CMT, and SBRT/NFV. Median delivered RT doses for CFRT and SBRT were 50.4 Gy in 1.8 Gy fractions and 35 Gy in 7 Gy fractions, respectively. TLCs from day 0 (the first day of RT) to 40 were recorded and analyzed using the Kruskal-Wallis test with *p*-values adjusted with Bonferroni’s method. Linear regressions were utilized to estimate the slope of TLCs as it changes with time and survival analysis was performed via Kaplan-Meier plots.

**Results:**

One hundred patients were identified (28 CFRT/CMT, 27 SBRT/CMT, 45 SBRT/NFV). Median pre-RT TLCs were not different among groups. Median lowest TLCs were significantly lower (*p* <  0.0001) and median TLCs reduction over time were significantly greater (*p* <  0.0001) in the CFRT group than SBRT groups. There was no difference in lowest TLCs or TLCs reduction over time between SBRT groups. Across all groups, the median time to lowest TLCs was similar. Survival analysis revealed no significant difference in median OS between SBRT and CFRT groups. However, in patients with surgery, Median OS for patients with SBRT/CMT was significantly higher than in those with SBRT/NFV (*p* = 0.03).

**Conclusions:**

Compared to CFRT, SBRT is associated with less lymphopenia. Further study of the effect of radiation technique on immune status is warranted.

## Background

Despite extensive research, adenocarcinoma of the pancreas remains one of the deadliest malignancies known to man. It is estimated that 55,440 patients will be diagnosed with pancreas cancer in 2018 with more than 44,000 ultimately succumbing to this disease [1]. With moderate clinical advances, the average 5 year overall survival rate of patients diagnosed with pancreas cancer has more than doubled in the past 2 decades though remains low at 8.5% for all patients and 31.5% in patients diagnosed with localized disease [2]. Unfortunately, more than 90% of patients are diagnosed with disease that has spread beyond the pancreas with more than half having evidence of distant metastatic disease at the time of diagnosis [2].

The role of radiation therapy in pancreatic adenocarcinoma remains somewhat controversial, though in the United States is primarily utilized in the settings of borderline-resectable or unresectable disease to provide either down staging to allow for future resection or improved local control, respectively. Traditionally, radiation therapy for the pancreas is provided to a relatively large volume including the primary tumor and regional lymphatics and is delivered over the course of 25–30 treatments [3, 4]. Importantly, conventionally fractionated radiation therapy (CFRT) is generally poorly tolerated with common toxicities including anorexia, nausea/vomiting, and diarrhea [5]. Additionally, past studies have indicated that CFRT is associated with iatrogenic lymphopenia [6, 7].

Reduction in total lymphocyte count (TLC) following radiation therapy has been previously shown to exist across multiple malignances including non-small cell lung cancer, glioblastoma, and squamous cell carcinoma of the head and neck with persistent association of treatment-associated lymphopenia with poor patient outcomes [8–11]. Indeed, treatment-associated lymphopenia has also been shown to reduce overall survival in patients treated with adjuvant radiation therapy for resected pancreas cancer as well as definitive chemoradiotherapy for patients with locally advanced disease [6, 7].

The etiology(s) underlying radiation-induced lymphopenia remain unclear. One proposed mechanism is that repeated irradiation of blood vessels near or within the treatment field allows the entire circulating blood pool to ultimately encounter meaningful radiation doses, thus damaging circulating lymphocytes as they flow through the blood stream near the treatment site [12]. Another proposed mechanism which has been studied specifically in pancreas irradiation is that inadvertent dose delivered to the spleen may account for the observed TLC reduction.^7^ Lastly, it is possible that treatment-induced lymphopenia is not directly related to the radiation treatment but rather to the concurrent chemotherapy often delivered during treatments for all of the malignancies thus far associated with this phenomenon. This latter mechanism is called into question, however, as if concurrent chemotherapy, which is often provided at a reduced dose, were to contribute significantly to treatment-associated lymphopenia it would be expected that full-dose induction chemotherapy would have similar effects though that was not shown to be the case in patients with either non-small cell lung cancer or locally advanced adenocarcinoma of the pancreas [7, 8].

Recently, the field of radiation oncology has started to see a paradigm shift in the treatment of pancreatic cancer with more clinics opting for short course stereotactic body radiation (SBRT) techniques in which high-dose radiation therapy is delivered over the course of 1–5 fractions [13]. Owing to the high dose per fraction delivered, treatment volumes for SBRT are often substantially reduced as compared to their CFRT counterparts, frequently focusing on the primary tumor alone. This technique is advantageous as it offers more convenience for the patient and is generally associated with low rates of acute toxicities, due mostly to the reduced treatment volumes. Importantly, it has previously been described that SBRT techniques result in significantly less treatment associated lymphopenia than CFRT and that greater TLC reduction resulted in worsened survival in patients treated with either radiation technique [14]. Based on this data and the proposed mechanisms for treatment induced lymphopenia, we sought to validate the association of SBRT with reduced treatment associated lymphopenia and determine if this translated into improvement in survival based on radiation technique. Further, we sought to further investigate the effects of systemic therapy delivered concurrent with radiation treatments on treatment-induced TLC reduction.

## Methods

### Patient selection

Medical records of patients with locally advanced pancreatic adenocarcinoma treated at the University of Nebraska Medical Center (UNMC) with neoadjuvant or definitive CFRT or SBRT following induction chemotherapy from 2004 to 2016 were retrospectively reviewed. In our study, locally advanced pancreatic disease was defined as superior mesenteric artery and/or celiac axis tumor encasement or superior mesenteric-portal vein confluence occlusion.

The following eligibility criteria were used to select the study population: (1) > 19 years of age, (2) Eastern Cooperative Oncology Group (ECOG) performance status ≤1, (3) biopsy-confirmed pancreatic adenocarcinoma, (4) radiology-confirmed locally advanced disease, (5) no previous abdominal irradiation, and (6) baseline (pre-radiation therapy) and follow-up complete blood counts accessible through the Electronic Patient Record.

As CFRT is commonly provided with concurrent systemic treatments but this practice is less consistent with SBRT, there was significant concern that alterations in concurrent treatment may ultimately confound analysis of iatrogenic alterations in lymphocyte counts. In an attempt to account for this, included CFRT patients were limited to those who received concurrent 5FU while SBRT patients were treated with concurrent 5FU or concurrent nelfinavir (an HIV protease inhibitor included in an institutional phase-I clinical trial as a potential radiation sensitizer). Through these groups, we were able to compare CFRT and SBRT provided with similar concurrent systemic therapy as well as various concurrent systemic therapies provided with SBRT in how each variable affects patient lymphocyte counts.

### Treatment and total lymphocyte counts

CFRT patients underwent computed tomography (CT) simulation in the supine position in custom-fitted immobilization devices with oral and intravenous contrast agents. The clinical target volume included the gross tumor volume plus regional lymphatic drainage areas and was expanded from 1.5 to 2.5 cm to generate the PTV. Radiation was delivered using either 3D conformal or intensity modulated techniques. The median CFRT prescription dose was 50.4 Gy (range 8–50.4Gy) at 1.8–2 Gy per fraction.

All SBRT patients underwent CT-guided implantation of up to 2 MRI-compatible fiducials placed within or near the pancreatic tumor. Patients underwent CT simulation in the supine position using custom-fitted immobilization devices with oral and intravenous contrast agents and 4-dimensional (4D) assessment of tumor motion. Median SBRT prescription dose was 35 Gy (range 25-40Gy) in 5 fractions.

Regardless of radiation technique, concurrent chemotherapy consisted of either 5FU administered by continuous infusion (2700 mg/m^2^/weekly) or capecitabine (800–1000 mg/m^2^ twice daily) taken Monday through Friday for a duration of between 30 and 39 days. SBRT concurrent Nelfinavir was administered at 1250 mg PO BID [15] for 3-5 weeks.

Total lymphocyte counts (TLCs) as available through routine complete blood counts were recorded from immediately prior to radiation delivery to day 40 (with day 0 corresponding to the delivery of the first fraction of radiation).

### Statistical analysis

Statistical comparisons were performed using the Kruskal-Wallis test with *p*-values adjusted using the Bonferroni method. Change in TLC levels over time were modeled through linear regression analysis. Kaplan-Meier plots were used for survival analysis. All statistical analysis was performed using SPSS version 20.0 and SAS software.

## Results

### Patients

In total, 100 patients were identified who met the inclusion criteria for this study. Of these, 28 received CFRT with concurrent chemotherapy (CFRT/CMT), 27 received SBRT with concurrent chemotherapy (SBRT/CMT), and 45 received SBRT with concurrent NFV (SBRT/NFV). Median age, gender distribution, tumor location, rates of subsequent resection, and baseline TLC levels were similar across groups (Table 1). Additionally, radiation doses were similar amongst the two groups receiving SBRT. Patients receiving CFRT/CMT tended to be diagnosed with lower-stage disease than those who underwent SBRT though stage at diagnosis was comparable between the two SBRT groups (Table 1).

**Table 1:** Clinical characteristics of the study population

Parameter	CFRT/chemo 28	SBRT/Chemo 27	SBRT/NFV 45	P1-value^d^	P2-value^d^
Age (years)				0.172^a^	
Median (range)	61 (49–76)	66 (43–96)	62 (34–79)		
Gender				0.197 ^b^	
male	12 (43%)	15 (56%)	29 (64%)		
female	16 (57%)	12 (44%)	16 (36%)		
Tumor location				0.080 ^b^	
Head/neck	25 (89%)	21 (78%)	43 (96%)		
Body/tail	3 (11%)	6 (22%)	2 (4%)		
Tumor Stage				0.029^b^	0.088^b^
IIA	16 (57%)	9 (33%)	14 (31%)		
IIB	4 (14%)	11 (41%)	13 (29%)		
III	8 (29%)	5 (19%)	18 (40%)		
IV	0 (0%)	2 (7%)	0		
Subsequent Resection					
yes	9 (32%)	10 (37%)	13 (29%)	0.763 ^b^	
no	19 (68%)	17 (63%)	32 (71%)		
Chemotherapy					
5FU or Capecitabine	28 (100%)	27 (100%)	0		
Nelfinavir	0	0	45 (100%)		
Dose/fractionation					
1.8Gy*13 = 23.4Gy	1 (3.6%)				
1.8Gy*17 = 30.6Gy	1 (3.6%)				
1.8Gy*28 = 50.4Gy	20 (71.4%)				
2.0Gy*4 = 8Gy	1 (3.6%)				
2.0Gy*25 = 50Gy	4 (14.2%)				
2.5Gy*17 = 42.5Gy	1 (3.6%)				
5GY*5		6 (22%)	8 (18%)		
6GY*5		4 (15%)	3 (7%)		
7GY*5		17 (63%)	9 (20%)		
8GY*5		0	25 (55%)		
Baseline TCLs (X 10^3^/µl)				0.740 ^a^	
Median (range)	1.48 (0.5–2.8)	1.43 (0.4–3.3)	1.38 (0.3–2.6)		
Lowest TCLs (X 10^3^/µl)				< 0.0001 ^c^	1.000^c^
Median (range)	0.29 (0–1.6)	0.74 (0.1–1.5)	0.68 (0–1.6)		
Days to Lowest TCLs				0.281 ^c^	
Median (range)	29 (13–38)	25 (0–40)	21 (0–40)		
Slope of TCLs Change (X 10^3^/µl per day)				< 0.0001^c^	0.242^c^
Median (range)	−0.021 (−0.05–0.02)	−0.012 (−0.09–0.01)	−0.008 (−0.03–0.04)		

^a^P was calculated from an ANOVA test Bonferroni adjustment;

^b^P was calculated from a Chi^2^ test or an Exact test;

^c^P was calculated from a Kruskal-Wallis test with Bonferroni adjustment;

^d^P1 for three sample comparisons and P2 for two SBRT sample comparisons

### Alteration of TLCs following treatment

**Fig. 1:**
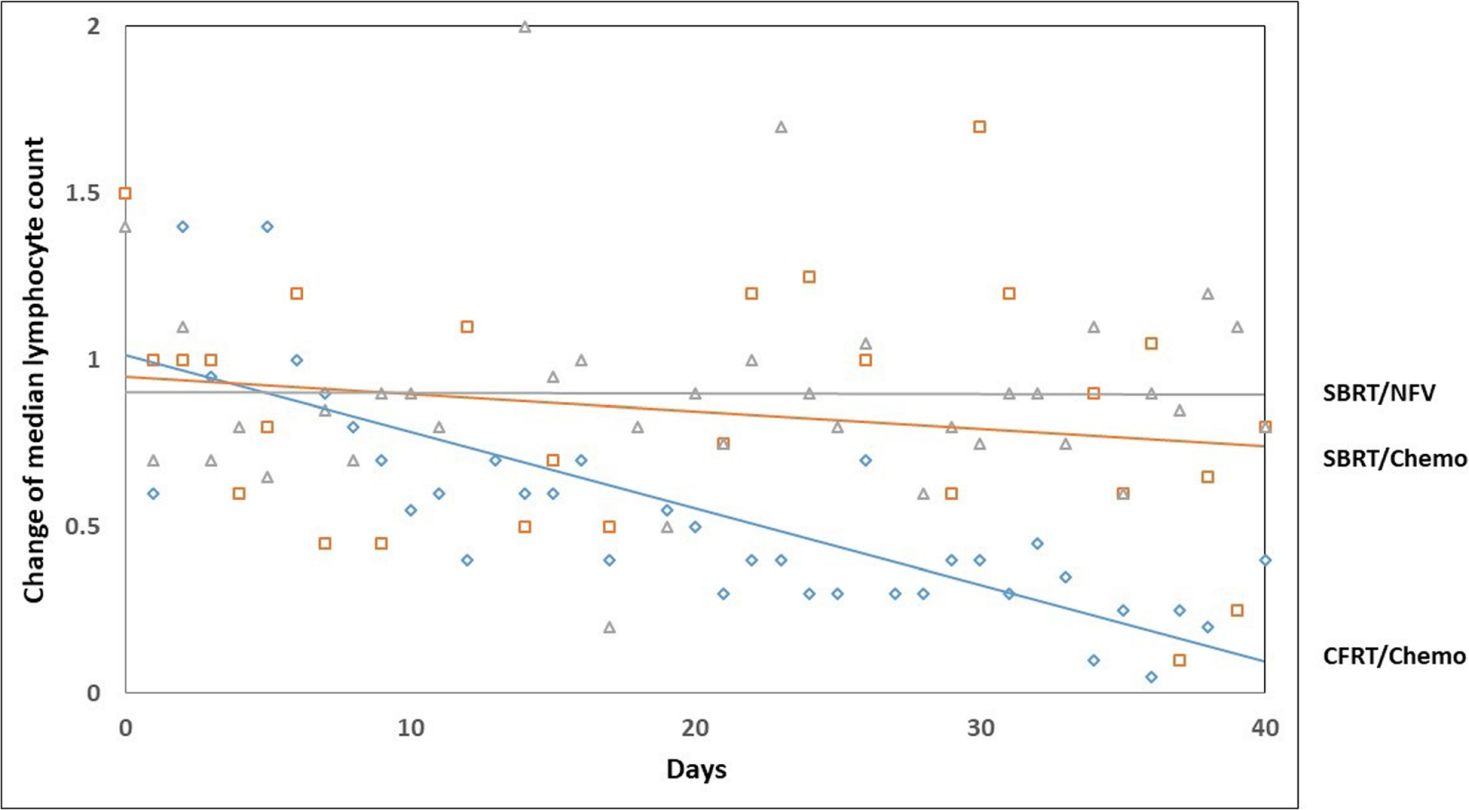
Median (95% CI) TLCs reduction over time in patients treated with CFRT/CMT (Blue), SBRT/CMT (orange), and SBRT/NFV (gray)

Iatrogenic reduction in TLC levels was noted in patients treated with CFRT/CMT, SBRT/CMT, and SBRT/NFV. The greatest trend for reduction in TLCs was noted in patients treated with CFRT/CMT (slope = (−)0.021) with those treated with SBRT/CMT and SBRT/NFV having a less pronounced TLC reductions (slope = (−)0.012 and (−)0.008, respectively; *p* <  0.0001)) (Table 1, Fig. 1).

**Fig. 2:**
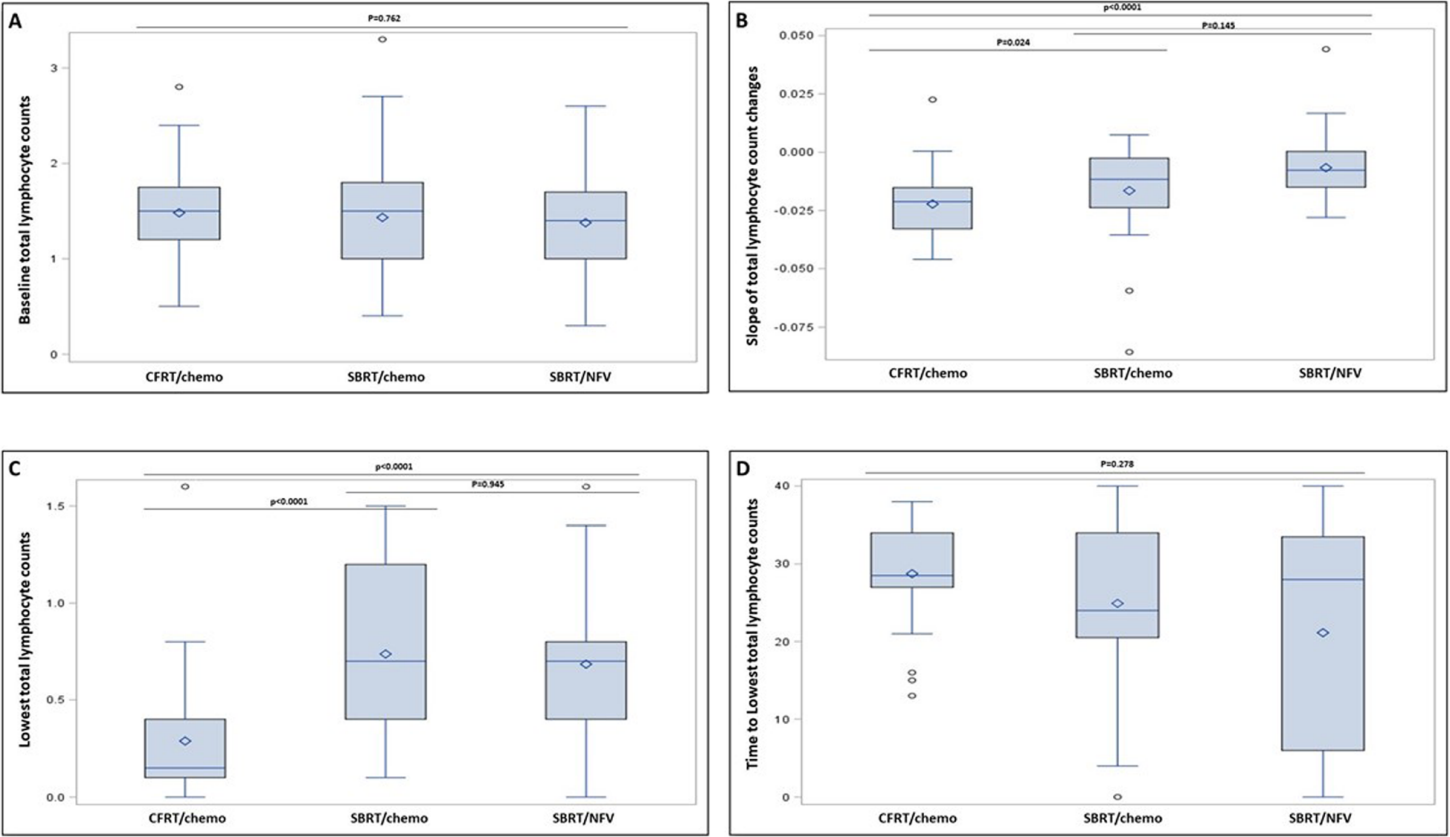
**a** Median (95% CI) pre-RT baseline TLCs (X 10^3^/µl), **b** Slope of median (95% CI) TLCs reduction over time (X 10^3^/µl per day), **c** Median (95% CI) lowest TLCs (X 10^3^/µl), and **d** Median (95% CI) time to lowest TLCs (days) in patients treated with CFRT/CMT, SBRT/CMT, and SBRT/NFV

Similarly, the lowest median TLC values recorded (95% confidence interval (CI)) following radiation were significantly lower in the CFRT/CMT group (0.29 (0–1.6)) than in the SBRT/CMT (0.74 (0.1–1.5)) or SBRT/NFV (0.68 (0–1.6)) groups (*p* <  0.0001) (Table 1, Fig. 2). Amongst the patients receiving SBRT, the lowest median TLC values were not different regardless of concurrent therapy (*P* = 1.000). The median time to realization of the lowest TLC value recorded did not vary across groups at 29, 25, 21 days, respectively (*p* = 0.281).

### Overall survival

**Fig. 3:**
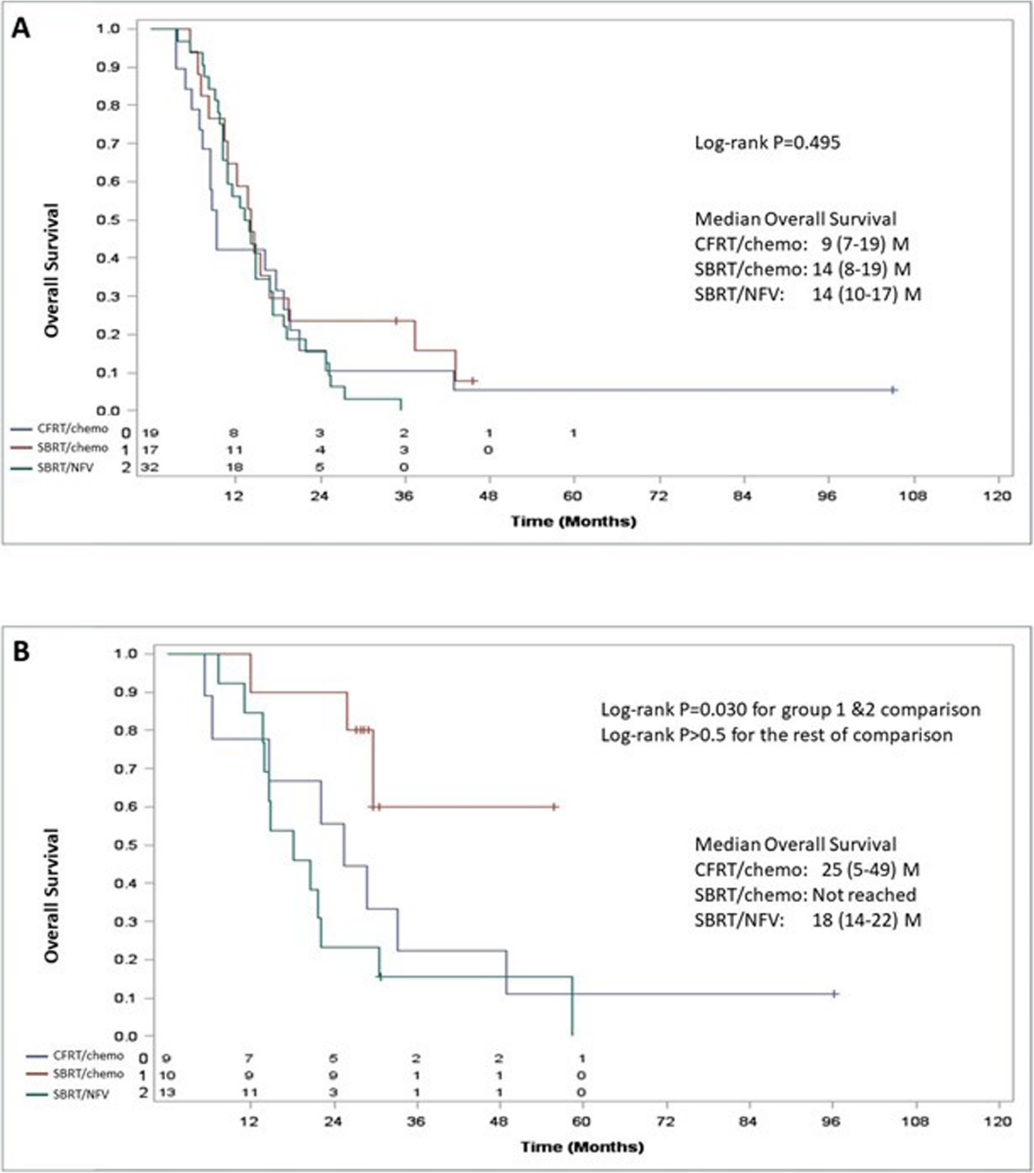
**a** Overall survival curves for patients treated with CFRT/CMT (Blue), SBRT/CMT (Red), and SBRT/NFV (Green), **b** Overall survival curves for patients treated with surgical resection following CFRT/CMT (Blue), SBRT/CMT (Red), and SBRT/NFV (Green)

Survival from diagnosis did not significantly differ between patients who received CFRT/CMT and those who were treated with SBRT/CMT or SBRT/NFV with median times to death (95% CI) of 9 (7–19) months, 14 (8–19) months, and 14 (10–17) months, respectively (*p* = 0.495, Fig. 3a). For those who underwent surgical resection following completion of radiation therapy, treatment with SBRT/CMT was significantly associated with longer overall survival than SBRT/NFV (*p* = 0.030) and showed a non-significant trend to improved survival compared to those treated with CFRT/CMT (Fig. 3b).

## Discussion

Similar to the previously published work by Wild et al., this current study indicates that SBRT for pancreas adenocarcinoma is associated with significantly less treatment-associated lymphopenia than CFRT, regardless of if the patient received any concurrent systemic therapy [14]. These results coincide well with the proposed mechanisms for treatment-induced lymphopenia resulting from dose to the blood-pool and/or spleen as the reduced treatment volume in SBRT would result in less dose to the spleen and great vessels and the reduction in fraction number would reduce the total volume of blood exposed to radiation over the treatment course [7, 12]. Importantly, though, treatment with SBRT regimens did not fully prevent the post-treatment down trending of TLCs in the 40 days following radiation treatment as both SBRT groups studied also showed negative TLC slopes on serial blood counts following treatment. This data suggests that radiation-induced lymphopenia cannot be fully abated through the reduction of treatment volume and fraction number as provided by stereotactic techniques.

Interestingly, in contrast to previously published reports the greater degree of treatment-induced lymphopenia observed in patients undergoing CFRT did not translate into a significant reduction in overall survival. The trend toward decreased survival in SBRT patients treated with concurrent NFV as compared to concurrent chemotherapy despite similar post-radiation TLC reductions suggests that this difference is irrespective of iatrogenic alterations in lymphocyte counts. Additionally, the subjective difference between overall survivals between the SBRT groups insinuates that NFV either does not confer clinically relevant radiosensitization or that the effect of radiosensitizing doses of 5-fluorouracil provides further benefit outside of improving radiation efficacy. Importantly, the overall survivals reported in this study are similar to those published by other groups previously, further validating our data [6, 14].

Despite the lack of statistical significance in the overall survival analysis, the presented data remain important in both the field of radiation oncology and in the general treatment of pancreatic adenocarcinoma. This analysis further bolsters the trend toward increased utilization of SBRT for pancreas cancer as this modern technique appears to reduce the likelihood of the thus far poorly understood though likely important acute/subacute toxicity of treatment associated lymphopenia. Further, this data continues to suggest that, while radiation therapy is considered a focal treatment technique resulting in toxicity within the treatment field alone, the effects of radiation therapy can be systemic and radiation treatment volumes may need to be adjusted to account for this. Our data suggests that such volume reduction through SBRT indeed will reduce this systemic toxicity. With further study into this phenomenon of treatment associated lymphopenia as well as the current ongoing research investigating the utility of immunotherapy and pancreatic adenocarcinoma, it is also probable that this data will become increasingly important in the near future as induction of lymphopenia through radiation therapy will likely significantly reduce the immune system’s ability to be utilized in the treatment of this disease [16].

We acknowledge that this study comes with multiple limitations. Specifically, the retrospective nature of this analysis increases the likelihood for the presence of unaccounted for confounding factors which limited interpretation of the data. Further, despite the reasonable number of patients included in the study as a whole, each treatment group analyzed individually had low patient numbers, reducing the power necessary for more robust analysis and further increasing the potential for false positive/negative results. Additionally, while long-term lymphopenia has been shown in cancer patients, and that long term lymphopenia has been associated with higher mortality after chemoradiation therapy in multiple studies [6, 17, 18], our data set was truncated at 40 days post radiation therapy. Due to the continued downward trend noted in our linear regression analysis across all patient groups, it is quite possible that the difference in treatment associated lymphopenia may be greater with increased elapsed time and thus provide greater statistical significance. Unfortunately, due to multiple factors including patients being treated with further chemotherapy of different regimens after 40 days, patients being lost to follow-up, patients being treated by outside medical oncologists, and the general poor prognosis of this disease, radiation-associated TLC data could not be reliably obtained in our patient population past this 40 day period. Therefore, we are not able to address the survival impact of radiation (with chemotherapy or nelfinavir)-induced long term lymphopenia in our cohort. This is a major limitation of this study. Lastly, treatment volumes prescribed for each SBRT patient that were not described in this data set may significantly affect the associated toxicities, including treatment associated lymphopenia. The effect of this confounding variable may be limited as all patients included in the study were treated by a single radiation oncologist (Chi Lin), though the lack of a standardized approach for SBRT treatment volumes in this setting undeniably increases this potential.

## Conclusion

Our data suggests that SBRT is associated with a significant reduction in treatment-induced lymphopenia when compared to CFRT but, in contrast to previously published work, this did not translate to a survival benefit associated with the more modern technique. Interestingly, the combination of chemotherapy with SBRT is associated with improved overall survival in patients who go on to receive a resection when compared to SBRT in combination with Nelfinavir, suggesting that this may be a superior treatment regimen in this selected patient population. We believe that this data, in combination with data previously reported by other groups, warrants further validation in a prospective manner as well as provides evidence for a potentially significant confounding variable which should be taken into the account in current and future immunotherapy studies.

## Data Availability

The datasets used and/or analyzed during the current study are available from the corresponding author on reasonable request.

## Abbreviations

4D: 4-dimensional
5FU: Fluorouracil
CF: Conventionally fractionated
Cl: Confidence interval
CMT: Chemotherapy
ECOG: Eastern Cooperative Oncology Group
NFV: Nelfinavir
OS: Overall survival
RT: Radiation therapy
SB: Stereotactic body
TLC: Total lymphocyte count
UNMC: University of Nebraska Medical Center
